# Promoter of COR2-like gene is a stress inducible regulatory region in banana

**DOI:** 10.1007/s11248-024-00405-w

**Published:** 2024-09-01

**Authors:** Sanjana Negi, Nikita Mahashabde, Subham Bhakta, Sudhir Singh, Himanshu Tak

**Affiliations:** 1 Plant Biotechnology Section, Nuclear Agriculture and Biotechnology Division, Bhabha Atomic Research Centre, Trombay, Mumbai, 400085 India; 2 Homi Bhabha National Institute, Anushakti Nagar, Mumbai, 400094 India; 3 National Agri-Food Biotechnology Institute, Department of Biotechnology, Mohali, 140306 India

**Keywords:** *Musa*, *CO2-like*, Tobacco, Promoter, GUS, Codeinone reductase

## Abstract

A promoter is a crucial component in driving the expression of a transgene of interest for biotechnological applications in crop improvement and thus characterization of varied regulatory regions is essential. Here, we identified the promoter of *COR2-like* (*codeinone reductase-like*) from banana and characterized its tissue specific and stress inducible nature. *MusaCOR2-like* of banana is closely related to *COR2* and *CHR* (*chalcone reductase*) sequences from different plant species and contains signature sequences including a catalytic tetrad typical of proteins with aldo–keto reductase activity. Transcript level of *MusaCOR2-like* was strongly induced in response to drought, salinity and exposure of signaling molecules such as abscisic acid, methyl-jasmonate and salicylic acid. Induction of *MusaCOR2-like* under stress strongly correlated with the presence of multiple cis-elements associated with stress responses in the *P*_*MusaCOR2-like*_ sequence isolated from *Musa* cultivar Rasthali. Transgenic tobacco lines harbouring *P*_*MusaCOR2-like*_*-GUS* displayed visible GUS expression in vascular tissue of leaves and stem while its expression was undetectable in roots under control conditions. Exposure to drought, salinity and cold strongly induced GUS expression from *P*_*MusaCOR2-like*_*-GUS* in transgenic tobacco shoots in a window period of 3H to 12H. Applications of salicylic acid, methyl-jasmonate, abscisic acid and ethephon also activate GUS in transgenic shoots at different period, with salicylic acid and abscisic acid being the stronger stimulants of *P*_*MusaCOR2-like*_. Using *P*_*MusaCOR2-like*_*-GUS* fusion and expression profiling, the current study sheds insights into a complex regulation of *COR2-like*, one of the least studied genes of secondary metabolite pathway in plants.

## Introduction

Biotechnology has revolutionized the field of plant science resulting in immense progress in crop improvement. Development of a transgenic crop requires a considerable understanding of regulatory elements in a plant specific manner as the regulatory mechanisms generally differs in various species. Hence, efforts are required to identify and characterize promoters for their tissue specific and inducible nature. Banana is an economically important crop and is extremely sensitive to various stress conditions. The availability of stress inducible promoters will assist researchers in genetic engineering of banana for modulation of its biochemical and physiological aspects. Banana contains numerous secondary metabolites such as alkaloids, flavonoids, terpenoids, glycosides, phenolics and tannins among others (Onyema 2016; Zaini 2022). Codeinone reductases (COR), are important enzymes of aldo–keto reductase (AKR) superfamily and are involved in NADPH-dependent reduction of carbonyl groups in key intermediates (Mindnich and Penning 2009). COR plays a crucial role in biosynthesis of pharmacologically important narcotics such as codeine and morphine in the opium plant (Unterlinner et al. 1999). In opium, six alleles of *COR* were identified and four cDNAs were reported as isoforms of COR with similar substrate specificity (Unterlinner et al. 1999). Members of the AKR superfamily have been identified in all the organisms and are classified into 18 subfamilies with functions in metabolite synthesis, detoxification and carbon assimilation (Penning et al. 2015). AKRs are thus involved in synthesis of many metabolites such as tropanes, cardiac glycosides, important alkaloids, flavonoids and ascorbic acid (Agius et al. 2003).

Many secondary metabolites are involved in stress responses and several of them including flavonoids, terpenoids, lignin and isoprenoids are induced in banana following infection of *Xanthomonas campestris pv. musacearum* (Tripathi et al. 2019). The antibacterial activity of banana leaf extract against methicillin-resistant *Staphylococcus aureus* had also been attributed to alkaloids and terpenoids (Sivasamugham et al. 2021). Jasmonic acid is a potent inducer of secondary metabolites including alkaloids in several plant species (Van der Fits and Memelink 2000). Further abiotic stresses such as salinity and drought have also been reported to positively influence the production of alkaloids in different plant species (Honório et al. 2021). However, cold stress has been documented to have a negative impact on production of alkaloids in *Catharanthus roseus* (Dutta et al. 2007). The regulation of different secondary metabolite genes especially those of alkaloid biosynthesis pathway under stress is poorly understood. Few reports on genome-wide identification of *AKRs* and their expression under stress have been documented in *Medicago truncatula* and *Solanum lycopersicum* indicating their importance in stress tolerance of plants (Yu et al. 2020; Guan et al. 2023). Reports on the beneficial effect of *AKR* overexpression on abiotic stress tolerance have also emerged confirming their diverse roles in various pathways of plant species such as peach, rice and *Physcomitrella patens* (Chen et al. 2019; Kanayama et al. 2014; Turoczy et al. 2011).

The regulation of *CORs* and their homologues under stress and inducible activity of their promoter regions in plants are largely unknown. Moreover, lack of adequate studies on promoter of secondary metabolism pathway genes have limited their applicability and hampered understanding of their compartmentalization in plants. Using promoter characterization, Dastmalchi and Dhaubhadel (2015) identified organs-specific expression of *chalcone isomerase* (*CHI*) genes in soybean. Bell-Lelong et al. (1997) characterized the tissue specific nature of *cinnamate-4-hydroxylase* (*C4H*) and correlated various cis-elements with its condition-dependent inducibility. However, only few studies on the promoter region of phenylpropanoid pathway genes such as *CHI*, *F3′5′H* and *C4H* have emerged providing clues on tissue specific and organ specific expression pattern of such genes (Negi et al. 2021, 2023; Van Tunen et al. 1990).

While screening multiple genes for transcript abundance in banana, a homologue of *COR, MusaCOR2-like* which is rapidly induced under stress conditions was identified. To gain further insights, we characterized the regulatory region of *MusaCOR2-like* for its tissue-specific and stress-induced activation to gain information on its possible role in the regulating the alkaloid biosynthesis during stress. The sequence of *MusaCOR2-like* contains signature sequences typical of *AKRs* and *COR* and displays similarity with other *AKRs* such as *CHRs*, *CORs*, *DMASs* and *MGRs*. Drought and salinity strongly induced *MusaCOR2-like* in the leaves of two important banana cultivars. Moreover, phytohormones such as ABA, SA and MeJA induce strong transcriptional activation of *MusaCOR2-like* in banana plants. The sequence of Promoter-*MusaCOR2-like* (*P*_*MusaCOR2-like*_) isolated from banana contains multiple cis-regulatory elements involved with varied stress responses. It displays an overabundance of GT1CONSENSUS and WRKY71OS associated with salicylic acid dependent expression and defense responses. Further, transgenic tobacco lines harboring *P*_*MusaCOR2-like*_*-GUS* were monitored for GUS expression to understand the tissue-specific expression and stress-mediated activation of *P*_*MusaCOR2-like*_ after exposures to drought, salinity, cold, ABA, MeJA, SA and ethephon.

Release of banana genome sequence has opened avenues toward understanding the genes and regulatory mechanisms associated with biochemical pathways which are least understood and require further detailed analysis (Droc et al. 2013). The current study will boost our knowledge on stress-mediated regulation of *CORs* and alkaloid biosynthesis pathways in plants. The present work also discusses the suitability of *P*_*MusaCOR2-like*_ in genetic manipulations and its potential applications in modulating alkaloid biosynthesis in plants.

## Material and methods

### Evolutionary relationship of *MusaCOR2-like* sequence

Complete open reading frame of *MusaCOR2-like* (Ma07_t12200.1) was fished from banana genome database (Droc et al. 2013) and a neighbour joining tree with 1000 bootstrap replications was constructed using closely related AKRs sequences imported from NCBI data base. Different protein sequences were aligned using clustal omega and the alignment file was processed to build the neighbour joining tree in MEGA6 software (Sievers et al. 2011; Kumar et al. 2008). AKR sequences used to build the phylogenetic tree were: *Arabidopsis thaliana* AtNOR (NP_176203.1), *Arabidopsis lyrata* AlCOR2X1 (XP_020890650.1), *Arabidopsis thaliana* AtAKR (AAK96820.1), *Glycine max* GmNDCS (XP_003547552.1), *Glycine max* GmMGR (XP_003534120.1), *Glycine max* GmCHR (NP_001353933.1), *Glycine max* GmCHR1 (NP_001235973.1), *Papaver somniferum* PsCOR2 (XP_026381711.1), *Pueraria candollei* PcCHR (QHF16705.1), *Sesbania rostrata* SrCHR (CAA11226.1), *Zingiber officinale* ZoCOR2 (XP_042447977.1), *Manihot esculenta* MeCOR2 (XP_021614369.1), *Citrus sinensis* CsCOR2 (XP_006483763.1), *Elaeis guineensis* EgCOR2 (XP_010932505.1), *Hevea brasiliensis* HbCOR2 (XP_021672109.1), *Populus euphratica* PeCOR2 (XP_011022103.1), *Elaeis guineensis* EgDMAS (XP_010936841.1), *Phoenix dactylifera* PdDMAS (XP_008798158.2), *Asparagus officinalis* AoCOR2 (XP_020244078.1), *Mangifera indica* MiCOR2 (XP_044490424.1), *Jatropha curcas* JcCOR2 (XP_012092025.1), and *Corchorus olitorius* CoAKR (OMO50812.1). Conserved signature sequences of AKRs and CORs in *MusaCOR2-like* were identified using multiple sequence alignment carried out using MEGA6 and GeneDoc software (Nicholas and Nicholas 1997). Sequences used with *MusaCOR2-like* to build the multiple sequence alignment were AoCOR2 (XP_020244078.1), ZoCOR2 (XP_042447977.1), EgCOR2 (XP_010932505.1), SrCHR (CAA11226.1), GmCHR (NP_001353933.1) and PsCOR2 (XP_026381711.1).

### Transcript profiling of *MusaCOR2-like*

*Invitro* cultures of banana cultivars Grand Naine, Rasthali and Karibale Monthan were grown and hardened in greenhouse conditions for two months. Uniform banana plants were subjected to salinity treatment (250 mM NaCl) and drought (dehydrating on filter paper) for definite time intervals (upto 24H). Plants were treated with phytohormones by spraying the solution on leaves. In separate experiments, SA (2 mM), MeJA (200 μM) and ABA (100 μM) were applied and leaf samples were collected and total RNA was isolated (Tak et al. 2021). Stress treatments and period of stress to plants were followed as reported in prior studies (Shekawat et al. 2011a; Shekhawat et al. 2011b; Tak et al. 2021). RNA was isolated with aid of Concert Plant RNA Reagent (Invitrogen) and RNeasy Plant RNA Isolation Mini Kit (Qiagen) and RNA free of genomic DNA contamination was converted to cDNA using reverse transcriptase enzyme (Thermoscript AMV-RT; Invitrogen). Quantitative RT-PCR analysis of *MusaCOR2-like* (FP: 5’-GGGTTCGACTGGGACTCC-3’ and RP: 5’-CCAGTGACTTGTAAGGGCC-3’) was performed using 1/10 diluted cDNA and SYBR green chemistry in a rotor gene-Q instrument (Qiagen). For normalization of transcript level between samples, expression of *EF1α* gene (FP:5’-CCGATTGTGCTGTCCTCATT-3’ and RP: 5’-TTGGCACGAAAGGAATCTTCT-3’) was also monitored and data was analysed as per the comparative Ct-method (2^−ΔΔCt^) (Schmittgen & Livak 2008).

### Isolation of ***P***_***MusaCOR2-like***_ and its sequence analysis

Primers specific to *P*_*MusaCOR2-like*_ were used and *P*_*MusaCOR2-like*_ was amplified from the genomic DNA of banana cultivar Rasthali. PLACE (Plant Cis-acting Regulatory DNA Elements) software (https://www.dna.affrc.go.jp/PLACE/?action=newplace) was used to scan the *P*_*MusaCOR2-like*_ for the presence of cis-regulatory elements associated with stress responses, hormonal signaling and tissue-specific expression.

### Generation of ***P***_***MusaCOR2-like***_-GUS fusion

*P*_*MusaCOR2-like*_ was sequenced and cloned upstream of *GUS* in *pCAMBIA1301* in *Hind*III and *Nco*I digestion sites. Sequences of primers used were: FP: 5’-AA*AAGCTT*TAATTGCTGTGCCAAAATGA-3’ and RP: 5’-AA*CCATGG*CTCTAAGCCGATCCAACACA-3’ *Hind*III and *Nco*I sites are italicized). Recombinant vector *pCAMBIA1301*-*P*_*MusaCOR2-like*_-*GUS* was confirmed by sequencing and then mobilized into *Agrobacterium tumefaciens* strain EHA105by electroporation.

### Transformation of tobacco with ***P***_***MusaCOR2-like***_***-GUS***

*“Nicotiana tabacum* L. cv. Havana 425*”* (NCBI: txid4097) was grown under in vitro conditions as per standard protocols. Leaf discs (1 cm^2^) of tobacco plants were punched and then co-cultured with acetosyringone (100 µM) induced recombinant EHA105. Infected leaf discs were then blotted on a filter paper and cultured on plain MS medium for two days in dark. The leaf discs were then transferred on regeneration medium containing MS-medium with BAP (1 mg/L), NAA (0.1 mg/L), 300 mg/L Cefotaxime, 300 mg/L Timentin and 20 mg/L Hygromycin. Shoots regenerated were cultured separately for further analysis.

### Assessment of ***P***_***MusaCOR2-like***_-***GUS*** activity

*P*_*MusaCOR2-like*_-*GUS* activation was monitored by histochemical staining of GUS activity. Explants were immersed in GUS staining solution (1 mM X-Gluc, 500 µM each of potassium ferrocyanide and potassium ferricyanide, 0.1% triton X-100) and staining was carried out at 37 °C for around 24H. GUS stained explants were visualized and recorded in a stereomicroscope equipped with camera (ZEISS Stemi 508; Carl Zeiss, USA). For stress-mediated activation of *P*_*MusaCOR2-like*_-*GUS,* different treatments were given to transgenic lines for different time period (1H, 3H, 6H, 12H, 24H). Drought, 250 mM salinity, 100 µM ABA, 2 mM SA, 200 µM MeJA and 5 mM ethephon were applied to transgenic lines and the GUS staining was then carried out (Tak et al. 2021).

### Statistical analysis

Each experiment comprised of minimum three biological replicates having three technical replicates. Normal distribution of the data sets was confirmed by performing the Shapiro–Wilk normality test in the Graphpad Prism software. Data sets having equal variance were analysed by Student’s t-test. Data sets showing normal distribution but having unequal variances were analysed with Welch’s t-test with a statistical significance level of 0.05. Deviations with *P* ≤ 0.05 from control values were considered as statistically significant differences. Statistically significant differences were denoted on the bars in the graphs with asterisk as per the standard notations (ns *P* > 0.05, *P ≤ 0.05, **P ≤ 0.01, ***P ≤ 0.001).

## Results

### Evolutionary relationship of *MusaCOR2-like*

**Fig. 1:**
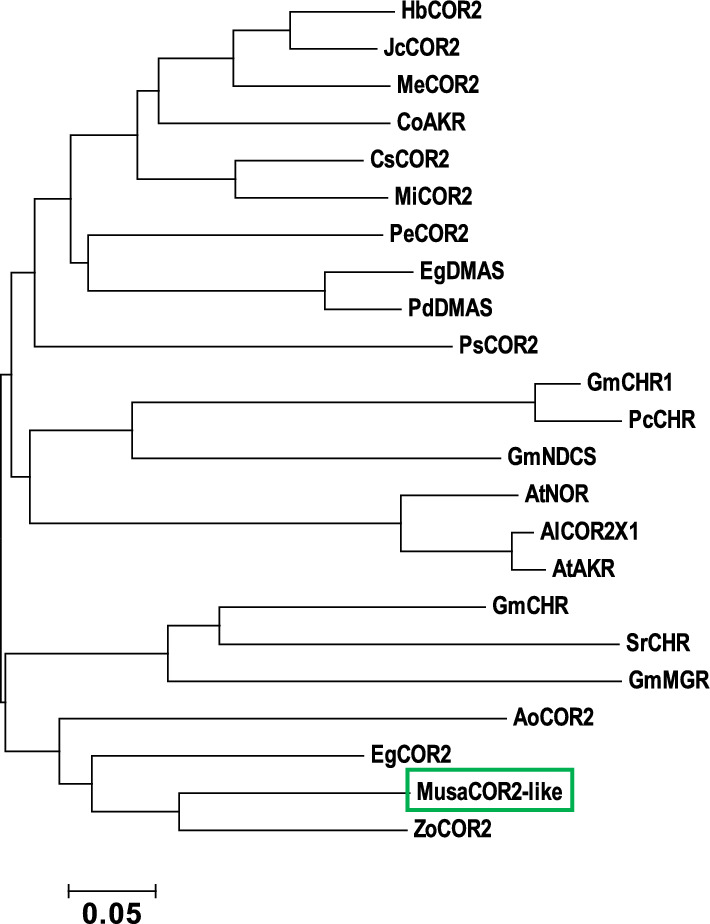
Evolutionary relationship analysis of *MusaCOR2-like* (Ma07_t12200.1) using a phylogenetic tree. Protein sequences included in the phylogenetic tree are *Arabidopsis thaliana* AtNOR (NP_176203.1), *Arabidopsis lyrata* AlCOR2X1 (XP_020890650.1), *Arabidopsis thaliana* AtAKR (AAK96820.1), *Glycine max* GmNDCS (XP_003547552.1), *Glycine max* GmMGR (XP_003534120.1), *Glycine max* GmCHR (NP_001353933.1), *Glycine max* GmCHR1 (NP_001235973.1), *Papaver somniferum* PsCOR2 (XP_026381711.1), *Pueraria candollei* PcCHR (QHF16705.1), *Sesbania rostrata* SrCHR (CAA11226.1), *Zingiber officinale* ZoCOR2 (XP_042447977.1), *Manihot esculenta* MeCOR2 (XP_021614369.1), *Citrus sinensis* CsCOR2 (XP_006483763.1), *Elaeis guineensis* EgCOR2 (XP_010932505.1), *Hevea brasiliensis* HbCOR2 (XP_021672109.1), *Populus euphratica* PeCOR2 (XP_011022103.1), *Elaeis guineensis* EgDMAS (XP_010936841.1), *Phoenix dactylifera* PdDMAS (XP_008798158.2), *Asparagus officinalis* AoCOR2 (XP_020244078.1), *Mangifera indica* MiCOR2 (XP_044490424.1), *Jatropha curcas* JcCOR2 (XP_012092025.1), and *Corchorus olitorius* CoAKR (OMO50812.1)

**Fig. 2:**
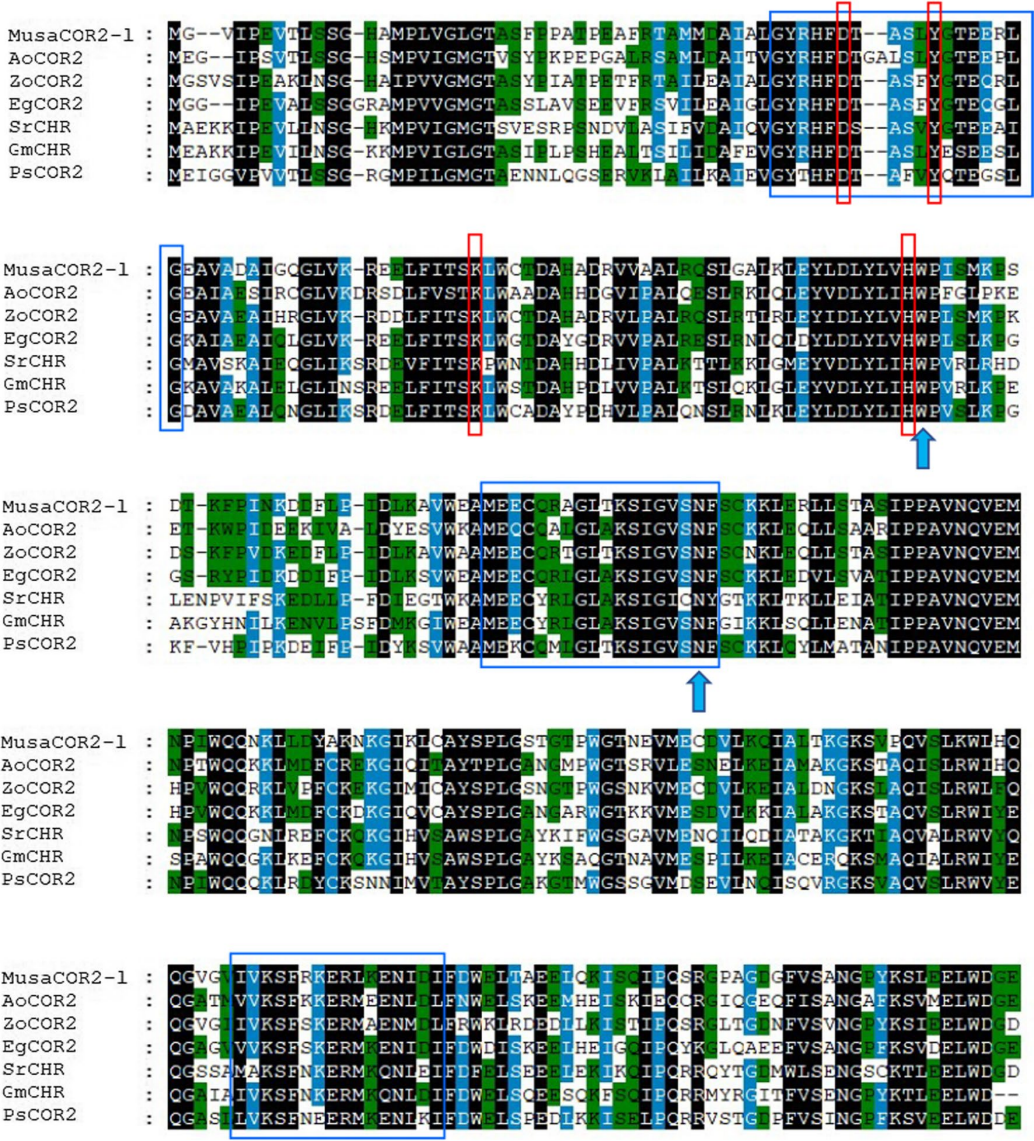
Sequence alignment of *MusaCOR2-like* with other AKRs. Alignment of *MusaCOR2-like* sequence with sequences of *Asparagus officinalis* AoCOR2 (XP_020244078.1), *Zingiber officinale* ZoCOR2 (XP_042447977.1), *Elaeis guineensis* EgCOR2 (XP_010932505.1), *Sesbania rostrata* SrCHR (CAA11226.1), *Glycine max* GmCHR (NP_001353933.1) and *Papaver somniferum* PsCOR2 (XP_026381711.1). Sequences within blue boxes are signatures typically observed in proteins with aldo–keto reductase activities. The four amino acids considered as AKRs catalytic tetrad are indicated by red boxes. Additional polar residues considered to be part of the catalytic site of AKRs are shown by blue arrows

Complete coding sequence of *MusaCOR2-like* contains 957 bp and codes for a 318 amino acids long protein having a theoretical pI of 5.67 and a predicted molecular weight of 35 kDa. Phylogenetic tree analysis suggested that *MusaCOR2-like* have a close homology with related AKR sequences such as EgCOR2 (XP_010932505.1), ZoCOR2 (XP_042447977.1) and AoCOR2 (XP_020244078.1). *MusaCOR2-like* and its closely related AKRs (EgCOR2, ZoCOR2 and AoCOR2) also have a close resemblance with CHR sequences of *Glycine max* (GmCHR; NP_001353933.1) and *Sesbania rostrata* (SrCHR; CAA11226.1) (Fig. 1). Three signature motifs (boxed in blue) typically present in AKR proteins such as COR2 of *Papaver somniferum* and CHRs were identified in *MusaCOR2-like* when aligned with GmCHR (NP_001353933.1), SrCHR (CAA11226.1), ZoCOR2 (XP_042447977.1), EgCOR2 (XP_010932505.1) and PsCOR2 (XP_026381711.1) (Fig. 2).Furthermore, a catalytic tetrad (boxed in red) comprising of Asp-53, Tyr-58, Lys-87, and His-120 commonly found in AKRs was also detected in *MusaCOR2-like* (Fig. 2). Besides, the catalytic tetrad two more polar residues (blue arrows), Trp-121, and Asn-167 considered to be important part of the catalytic site of AKRs were also detected in *MusaCOR2-like* (Fig. 2).

### Transcript profiling of *MusaCOR2-like*

**Fig. 3:**
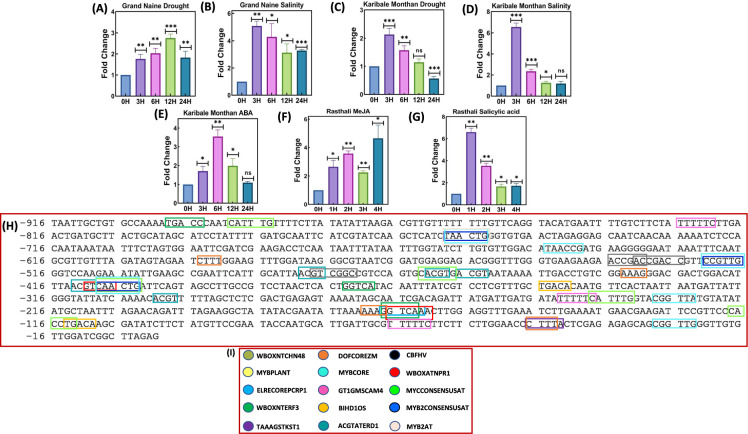
Quantitative RT-PCR analysis of *MusaCOR2-like* and detection of cis-regulatory elements. **A** Expression of *MusaCOR2-like* at different time points in leaves of banana cultivar Grand Naine exposed to drought. **B** Transcript abundance of *MusaCOR2-like* in leaves of banana cultivar Grand Naine during salinity stress. **C**–**E** Expression *MusaCOR2-like* in leaves of banana cultivar Karibale Monthan exposed to drought, salinity and ABA exposure respectively. **F** and **G** Analysis of transcript abundance of* MusaCOR2*-*like* in leaves of *Musa* cultivar Rasthali exposed to methyl jasmonate (MeJA) and salicylic acid respectively. **H** Cis-regulatory elements detected in a 916 bp upstream regulatory region of *MusaCOR2-like* using PLACE software. Cis-elements in *P*_*MusaCOR2-like*_ for responses to various stresses and tissue specific expression are boxed in colour. **I** Colour coding of different cis-element identified in *P*_*MusaCOR2-like*_ and boxed in (**H**). For quantitative RT-PCR of *MusaCOR2-like*, the fold change over control value was determined and expressed as mean ± SD

We analysed whether *MusaCOR2-like* responds to various environmental and physiological cues such as stress conditions and phytohormone presence in banana plants. Drought stress rapidly induced transcription of *MusaCOR2-like* in leaves of *Musa* cultivar Grand Naine which peaked at 12H then dropped towards 24H but remained higher than the control level (Fig. 3A). However, during salinity stress in *Musa* cultivar Grand Naine, the expression of *MusaCOR2-like* in leaves peaked around 3H and slowly reduced towards 24H post-stress (Fig. 3B). Drought and salinity stress strongly induced *MusaCOR2-like* in leaves of banana cultivar *Karibale Monthan* and transcript levels were maximum during 3H post stress and then gradually reduced towards 24H time point (Fig. 3C, D). ABA application also stimulated *MusaCOR2-like* expression and it peaked around 6H time point after which it returned to control level during 24H time point (Fig. 3E). Applications of MeJA and SA also rapidly induced transcription of *MusaCOR2-like* expression in leaves of *Musa* cultivar Rasthali suggesting important roles of *MusaCOR2-like* in stress conditions (Fig. 3F, G).

### Detection of *cis*-regulatory elements in the *P*_*MusaCOR2-like*_

A 916 bp upstream regulatory region of *MusaCOR2-like* was amplified from banana cultivar Rasthali and its sequence was analyzed for presence of cis-elements in PLACE software. *P*_*MusaCOR2-like*_ contains multiple cis-elements such as ACGTATERD1, CBFHV, ELRECOREPCRP1 and MYB2CONSENSUSAT among others which are known to be involved in varied stress responses involving drought, salinity, cold and applications of important signalling molecules like SA and ABA (Fig. 3H, I). Various cis-elements, their functions, conserved site and their locations within *P*_*MusaCOR2-like*_ are listed in Table 1.

**Table 1:** Cis-elements in the *MusaCOR2* promoter

No	Cis-element	Location in* MusaCOR2* promoter	Sequence	Function
1	ARR1AT	− 75, − 134, − 201, − 255, − 261, − 268, − 324, − 405, − 571, − 598, − 724, − 735, − 893	NGATT	Cytokinin associated expression
2	ACGTATERD1	− 480, − 462, − 457, − 301, − 413	ACGT	Drought and senescence mediated expression
3	ABREOSRAB21	− 480	ACGTSSSC	ABA associated expression
4	ABRELATERD1	− 463, − 462	ACGTG	Drought and senescence related expression
5	ASF1MOTIFCAMV	− 459, − 412	TGACG	Auxin and salicylic acid induced expression
6	AUXRETGA1GMGH3	− 459	TGACGTAA	Auxin responsive element
7	BIHD1OS	− 346, − 114	TGTCA	Pathogen induced expression
8	CBFHV	− 536, − 532, − 478	ATCGAC	cold associated expression
9	DOFCOREZM	− 595, − 434, − 171, − 47	AAAG	Binding site of Dof transcription factors
10	DPBFCOREDCDC3	− 658	ACACNNG	ABA responsiveness
11	DRE2COREZMRAB17	− 536, − 532	ACCGAC	ABA and drought responses
12	DRECRTCOREAT	− 536, − 532, − 478	RCCGAC	Cold and drought responses
13	ELRECOREPCRP1	− 168	TTGACC	Elicitor and salicylic acid responsiveness
14	EBOXBNNAPA	− 890, − 463, − 409, − 238, − 118	CANNTG	Tissue-specific induction of phenylpropanoid pathway genes
15	ERELEE4	− 153	AWTTCAAA	Ethylene responsive element
16	GT1GMSCAM4	− 826, − 243, − 67	GAAAAA	Pathogen inducibility and high salinity response
17	GT1CONSENSUS	− 67, − 142, − 243, − 312, − 364, − 826	GRWAAW	Salicylic acid inducible expression
18	LTRECOREATCOR15	− 439, − 478, − 535, − 531	CCGAC	ABA and cold responses
19	MYCCONSENSUSAT	− 890, − 463, − 409, − 238, − 118	CANNTG	Cold and ABA signalling associated element
20	MYB2CONSENSUSAT	− 759, − 522, − 409	YAACKG	Drought and abscisic acid signalling associated element
21	MYBCORE	− 759, − 645, − 522, − 409, − 229, − 29	CNGTTR	Dehydration response and flavonoid biosynthesis associated element
22	MYB2AT	− 759	TAACTG	Drought associated expression
23	OSE1ROOTNODULE	− 597	AAAGAT	Response for infection of root nodules
24	PALBOXAPC	− 473	CCGTCC	Consensus element of PAL gene
25	POLLEN1LELAT52	− 65, − 510, − 706, − 824, − 883	AGAAA	Element associated with expression in pollens
26	TAAAGSTKST1	− 47	TAAAG	Guard cell associated expression
27	T/GBOXATPIN2	− 302	AACGTG	Jasmonic acid and wound inducibility
28	WRKY71OS	− 899, − 459, − 411, − 373, − 346, − 167, − 114	TGAC	Defence responses
29	WBBOXPCWRKY1	− 168	TTTGACY	Elicitor associated response elements
30	WBOXNTERF3	− 899, − 374, − 168	TGACY	Wound associated expression
31	WBOXATNPR1	− 167, − 411	TTGAC	Salicylic acid associated expression

### Tissue-specific activity of ***P***_***MusaCOR2-like***_-GUS

**Fig. 4:**
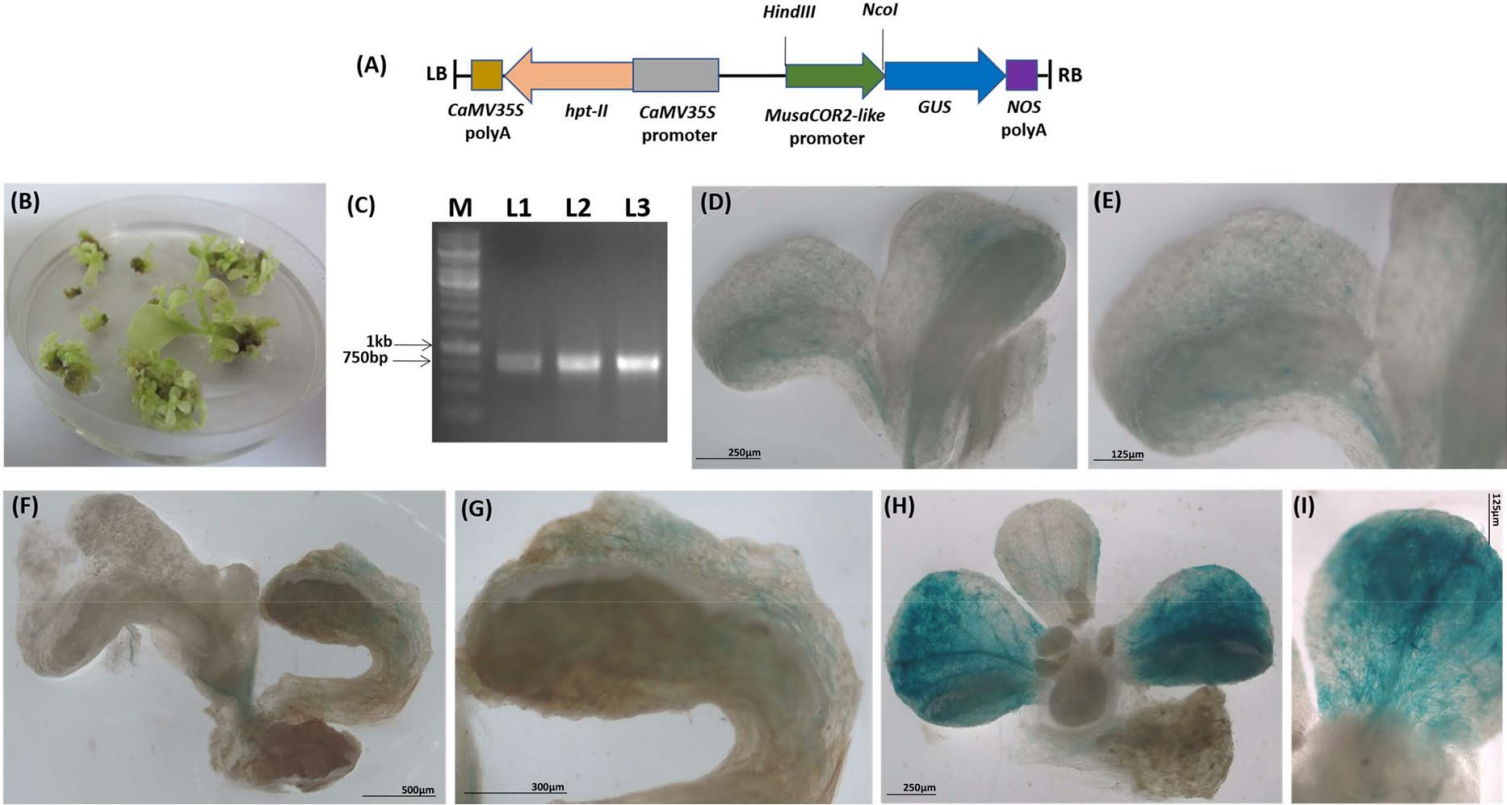
Tissue specific activity of *MusaCOR2-like* promoter in transgenic tobacco lines. **A** T-DNA region harboring* P*_*MusaCOR2-like*_ upstream of *GUS* in binary vector *pCAMBIA1301*. This T-DNA region also contains a cassette for expression of *hygromycin phosphotransferase* (*hpt-II*) gene in transgenic tobacco lines. **B** Emergence of putatively transformed plants from leaf discs of tobacco on hygromycin supplemented medium. **C** PCR based confirmation of T-DNA integration by amplification of *hpt-II* coding region from the genome of tobacco lines. (L1-L3: transgenic tobacco lines; M: 1 kb DNA ladder) Position of 1 kb and 750 bp bands in marker are indicated by arrow. **D** Expression of *GUS* under control of *P*_*MusaCOR2-like*_ in transgenic tobacco lines L1. **E** Close-up of L1 in (**D**) demonstrating the expression of GUS in vascular tissue of leaf. **F** and **G** GUS histochemical staining in line L2 showing the expression of *GUS* due to activation of *P*_*MusaCOR2-like*_ in vascular tissue. **H** GUS staining in transgenic line L3. **I** Close-up of leaf of line L3 in (**H**) showing intense GUS expression in leaves due to activity of *P*_*MusaCOR2-like*_

We fused *P*_*MusaCOR2-like*_ upstream of *GUS* in *pCAMBIA1301* and used an *Agrobacterium*-mediated method to regenerate tobacco lines harbouring *P*_*MusaCOR2-like*_-*GUS* (Fig. 4A). Leaf disc-based transformation through *Agrobacterium* and selection on Hygromycin supplemented medium resulted in many shoots putatively transformed with T-DNA harbouring *P*_*MusaCOR2-like*_-*GUS* (Fig. 4B). Three transgenic lines emerged from different leaf disc were randomly chosen for further analysis and were named as L1-L3. PCR amplification of a portion of *hygromycin phosphotransferase-II* (*hpt-II*) present within the T-DNA region from the genomic DNA of these tobacco lines confirmed the stable integration of T-DNA in these lines (Fig. 4C). GUS histochemical staining of L1-L3 indicated weak activation of* P*_*MusaCOR2-like*_ in vascular tissue of all the three lines under control conditions (Fig. 4D–I). The expression of *P*_*MusaCOR2-like*_-*GUS* in L1 and L2 appeared to be confined mainly to the vascular region (Fig. 4D–F) which become evident in the close-up images of L1 and L2 (Fig. 4E–G). In transgenic line L3, the *GUS* expression due to activity of *P*_*MusaCOR2-like*_-*GUS* was detected in vascular tissues and epidermal cells of a few leaves (Fig. 4H–I).

### Activation profiles of ***P***_***MusaCOR2-like***_-GUS under stress

**Fig. 5:**
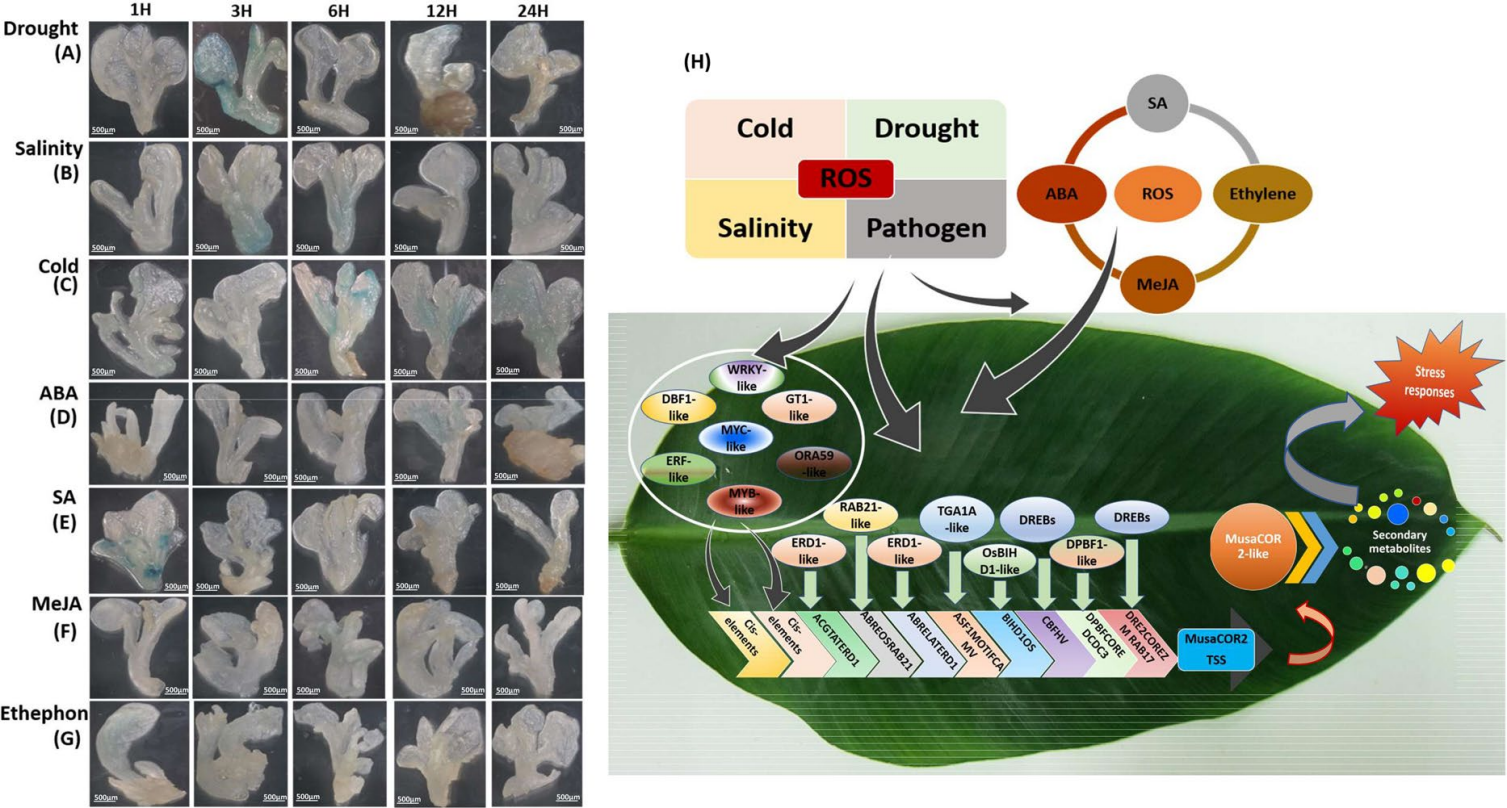
Response of *P*_*MusaCOR2-like*_ to stress and hormonal applications. Shoots of transgenic tobacco lines harboring *P*_*MusaCOR2-like*_-*GUS*were exposed to different conditions and *P*_*MusaCOR2-like*_*-GUS* activation was monitored at different time points by GUS staining. **A**–**G** Demonstrative GUS staining profiles at 1H, 3H, 6H, 12H and 24H of **A** drought, **B** salinity, **C** cold, **D** ABA, **E** SA, **F** MeJA and **G** ethephon treatment. The experiments were performed in triplicate and data of one replication is shown for demonstration purpose. **H** Probable mechanism of *P*_*MusaCOR2-like*_ regulation during stress in banana plants. Stress and signalling molecules through molecular pathways trigger various transcription factors. These transcription factors then bind to various cis-elements in *P*_*MusaCOR2-like*_ and regulate its transcription. Some of the cis-elements in *P*_*MusaCOR2-like*_ and their probable binding factors are depicted. Elevated *MusaCOR2-like* modulates secondary metabolites having potential roles in stress responses. *SA* salicylic acid, *MeJA* methyl jasmonate, *ROS* reactive oxygen species, *ERD1* early response to dehydration1, *RAB21* responsive to ABA, *TGA1A* TGACG-binding transcription factor, *DREB* dehydration-responsive element-binding protein, *DPBF1* Dc3 promoter-binding factor-1, *DBF1* DRE binding factor 1

As transcript levels of *MusaCOR2-like* were strongly altered under stress and influence of phytohormones, we analyzed the activity of *P*_*MusaCOR2-like*_-*GUS* in transgenic lines under stress conditions at different time points (1H, 3H, 6H, 12H and 24H). Drought imposition strongly activated *GUS* expression from *P*_*MusaCOR2-like*_ at 3H time point which then gradually reduced towards 24H post drought exposure (Fig. 5A). High-salinity stress also activated *P*_*MusaCOR2-like*_-*GUS* after 3H period which remained stronger till 6H and then returned back to the control level around 24H period (Fig. 5B). During cold exposure, GUS expression was strongly activated around 6H and then reduced towards 24H but remained higher than the control level (Fig. 5C). Application of ABA to transgenic lines did not alter GUS expression till 6H but was strongly activated during 12H period after which it reduced towards 24H but remained stronger than the control level (Fig. 5D). Exposure of SA, activated *GUS* expression in transgenic lines from *P*_*MusaCOR2-like*_-*GUS* as early as 1H which then quickly returned to control level around 3H and remained there until 24H post application (Fig. 5E). MeJA appeared to weakly stimulate GUS in transgenic lines around 6H which at later time points returned to control level (Fig. 5F). Ethephon also weakly induced GUS expression in transgenic lines harbouring *P*_*MusaCOR2-like*_-*GUS* around 1H to 3H which then reduced towards 24H time frame (Fig. 5G). A pictorial representation of the probable regulation of *P*_*MusaCOR2-like*_ during stress and by phytohormones in banana plants is presented in Fig. 5H. Stress and phytohormones through various pathways activate various stress-related transcription factors such as ERD1-like, RAB21-like, TGA1A-like, DREB-like, DPBF1-like, DBF1-like, WRKY-like, MYC-like, ERF-like, MYB-like among others. *P*_*MusaCOR2-*_like harbours various cis-elements as binding sites for stress related regulatory factors and their binding can differentially regulate the transcription rate of *MusaCOR2-like* in condition dependent manner. Elevated *MusaCOR2-like* then regulates the biosynthesis of various secondary metabolite products having potential roles in stress response of banana plants.

## Discussion

Promoters having low activation in control condition and with strong induction under stress conditions are desirable to generate stress tolerant plants. Overexpression of genes under strong and constitutive promoters such as CaMV35S promoter or poly-ubiquitin promoter generally leads to unwanted excessive background expression deleteriously affecting growth and yield (Nakashima et al. 2007). Hence, in the current study, we functionally analyzed the 5’-regulatory region of *MusaCOR2-like* from banana plants and studied its tissue-specific nature and stress-dependent activation in tobacco plants. *MusaCOR2-like* has remarkable homology with multiple AKR proteins and displays properties of stress inducibility. The presence of critical residues such as catalytic tetrad (Asp-53, Tyr-58, Lys-87, and His-120) along with polar residues (Trp-121 and Asn-167) important for catalytic site of AKRs confirmed that *MusaCOR2-like* indeed is a AKR and has probable functions in physiological responses (Bomati et al. 2005). This is evident with close clustering of *MusaCOR2-like* with CHR sequences belonging to AKR superfamily (Bomati et al. 2005). Goormachtiget al. (1999) reported three consensus sequences typical of AKRs in CHR sequence of *Sesbania rostrata* and their presence in *MusaCOR2-like* further indicated its close association with AKRs. However, little is known about the *COR2-like* sequences and their activities in plant species other than *Papaver somniferum* are largely unknown (Unterlinner et al. 1999).

Although, expression of COR sequence in different organs of *Papaver somniferum* has been determined, little is known about the transcriptional regulation of *COR* sequences under stress. Moreover, no efforts on analysing the 5’-regulatory  region of *COR2-like* sequences have ever been carried out. Such studies can provide valuable information on the regulation of alkaloid biosynthesis of plants under stress. Some studies have recently emerged on analysis of the 5’-regulatory region of different genes involved in secondary wall deposition, stress responses and phenylpropanoid biosynthesis pathway in banana (Negi et al. 2018, 2021; Tak et al. 2021). Despite that, research on understanding the regulation of genes of secondary metabolism pathway at the level of promoter region is in infancy. Studies in this direction will provide clues on cross-talks between secondary metabolism and stress responses, eventually generating plants with superior agronomic traits. Towards this, we analysed the 5’-regulatory  region of *MusaCOR2-like* gene of banana and studied its tissue-specific nature and stress-inducible activity using transgenic tobacco lines. To the best of our knowledge, the regulation of COR genes under stress and its promoter activity have not been studied and reported.

Phenolics and secondary metabolites elevation during stress has been linked with improved tolerance in many plant species. Some studies had related stress and secondary metabolites accumulation in banana. Chitosan nanoparticles used to counter abiotic stress have been shown to increase the secondary metabolites content in leaves of banana by upto 41%, indicating stress protective roles of these metabolites (Wang et al. 2021).Significant alteration in the content of various secondary metabolites of broad classes, such as amines, ketones, propane, phenols, esters etc., was detected in banana shoots supplemented with a high dose of sodium chloride (Chen et al. 2022; Dikayani et al. 2019). Thus, similar to other plants, secondary metabolites accumulation is an integral mechanism of stress responses in banana also. Banana cultivar Williams showed relatively better cold tolerance than Grand Nain as it accumulated significantly more soluble carbohydrates and phenolics during an assay for 48 h at 5 °C, indicating a strong relation between secondary metabolites accumulation and stress response in banana (El-Mahdy et al. 2018). Transcriptome analysis of drought sensitive and tolerant banana cultivars has pointed to a significant enrichment in DEGs belonging to alkaloid biosynthesis, terpenoids biosynthesis and other secondary metabolites, indicating pivotal roles of these compounds in stress responses of plants (Muthusamy et al. 2016). Hence, elevation of secondary metabolites during stress is integral to stress responses in banana plants.

Variation of B-genome content is associated with alteration of stress tolerance in banana cultivars and the effect of B-genome copies on stress responses has also been experimentally analysed (Placide et al. 2012; Vanhove et al. 2012). In our experiment, we analysed whether expression of *MusaCOR2-like* is responsive in banana cultivars with varying B-genome content. Hence, we used three *Musa* cultivars, Grand Naine (AAA genome), Karibale Monthan (ABB genome) and Rasthali (AAB genome) and analysed the effect of B-genome on stress responsiveness of *MusaCOR2-like* promoter. Variation in transcript levels of *MusaCOR2-like* in these important cultivars suggest its possible functions in mitigation of stress symptoms and indicate stress responsiveness of this promoter in varying *Musa* cultivars. Drought remarkably induced *MusaCOR2-like* expression in leaves of banana cultivars Karibale Monthan and Grand Naine. Drought inducibility of *MusaCOR2-like* can be correlated with cis-elements such as ACGTATERD1, DRECRTCOREAT and MYB2AT among others within *P*_*MusaCOR2-like*_. Salinity is another strong inducer of *MusaCOR2-like* in banana and GT1GMSCAM4 element associated with salinity responses within *P*_*MusaCOR2-like*_ points towards potential roles of *MusaCOR2-like* in mitigating such stress. Drought and salinity are reported to induce *stylopine synthase,* a key enzyme of alkaloid biosynthesis which can be associated with augmented concentration of dihydrocoptisine under drought and salinity in *Chelidonium majus* (Yahyazadeh et al. 2018). Yahyazadeh et al. (2021) suggested that stress dependent elevation of alkaloids is primarily linked with elevated expression of genes rather than stress-associated passive shift. Increased transcription of *MusaCOR2-like* under drought and salinity supports the view of elevated genes expression rather than a passive shift as driving force for alkaloid accumulation. MeJA and SA strongly induced *MusaCOR2-like* which suggest its roles in defence responses of banana which is further supported by identification of ASF1MOTIFCAMV, ELRECOREPCRP1, GT1CONSENSUS, WBOXATNPR1 and T/GBOXATPIN2 regulatory elements within *P*_*MusaCOR2-like*_. Additionally, identification of defence and elicitation linked WRKY71OS and WBBOXPCWRKY1 in *P*_*MusaCOR2-like*_ points towards the roles of *MusaCOR2-like* in immune responses of banana plants. Moreover, MeJA and SA are known elicitors of secondary metabolites and a recent report documented their effect on reserpine and ajmalicine accumulation in *Rauvolfia serpentina* (Dey et al. 2020). Detection of ABA-related cis-elements such as ABREOSRAB21, DPBFCOREDCDC3, LTRECOREATCOR15 and MYCCONSENSUSAT in *P*_*MusaCOR2-like*_ was in line with induction of *MusaCOR2-like* in banana under ABA application. Our observation is supported by induction of alkaloids in *Catharanthus roseus* due to ABA application (Saenz et al. 1993). The RT-qPCR data of *MusaCOR2-like* in leaves of banana plants under stress conditions indicated the stress inducible nature of its promoter region and these observations can be further validated and strengthened by western blot analysis using anti *COR2-like* monoclonal antibodies.

*β-D-glucuronidase* (*GUS*) reporter gene had proved vital in characterization of temporal and inducible nature of promoter regions which has resulted in rapid increase in availability of regulatory resources for crop improvement. Through *Agrobacterium* mediated transformation, three transgenic tobacco lines (L1, L2 and L3) harbouring *P*_*MusaCOR2-like*_-*GUS* were regenerated and utilized to study the tissue-specific nature and stress-inducible activity of *P*_*MusaCOR2-like*_. GUS histochemical staining indicated weak activity of *P*_*MusaCOR2-like*_-*GUS* in vascular tissue of transgenic lines however, *GUS* expression was also detected in epidermal cells of some leaves in L3. Our observation is supported by studies reporting high accumulation of alkaloids and flavonoids in the xylem and phloem tissues such as that in case of *Sophora flavescens* (Wei et al. 2021; Nowak and Selmar 2016). Stress was then applied to transgenic lines to study the stress mediated activation of *P*_*MusaCOR2-like*_. Drought and salinity exposure strongly activated *P*_*MusaCOR2-like*_-*GUS* in transgenic lines which corroborates with the induction of *MusaCOR2*-*like* in banana under these conditions. Cold also activated *P*_*MusaCOR2-like*_-*GUS* in tobacco plants and cis-elements such as CBFHV, DRECRTCOREAT, LTRECOREATCOR15 and MYCCONSENSUSAT in *P*_*MusaCOR2-like*_ correlated with cold stress response of *MusaCOR2-like*. Differences in expression profiles of *MusaCOR2-like* in banana and activity of *P*_*MusaCOR2-like*_-*GUS* in tobacco under stress suggested genetic backgrounds linked differences in regulatory mechanisms. Such variations in the expression of genes had earlier been linked with either position effects or an outcome of dissimilarities in genetic backgrounds (Adamczyk Jr et al. 2009). Both SA and MeJA activated *GUS* expression from *P*_*MusaCOR2-like*_-*GUS* in tobacco lines and SA induced GUS expression was early and remarkably higher than MeJA. Superior activity of SA over MeJA on increasing alkaloids and other secondary metabolites have emerged supporting our observations (Chaichana and Dheeranupattan 2012). SA and MeJA are implicated in defense responses and their roles in activating *P*_*MusaCOR2-like*_-*GUS* in tobacco lines supported roles of *MusaCOR2-like* in defense responses of banana plants. Alkaloids are indeed reported as an important component in defense arsenal of plants against pathogen and herbivores (Reimann et al. 2004). *Fusarium Oxypsorum* is a dreaded pathogen for banana cultivation and alkaloids among other metabolites are reported to strongly accumulate in banana in response to *Fusarium* infection (Dong et al. 2020). Manipulation of *MusaCOR2-like* for altering alkaloids content can be an important approach to manage *Fusarium* infection. This is supported by a report on *Peganum harmala* where alkaloids inhibited *Fusarium oxysporum* through induction of cell death (Zhu et al. 2022). Single ERELEE4 in *P*_*MusaCOR2-like*_, an ethylene-responsive element and weak activation of *P*_*MusaCOR2-like*_-*GUS* in tobacco plants after ethephon application indicated ethylene signalling as another factor in controlling *MusaCOR2-like* expression. These observations align with reports on regulation of alkaloid biosynthesis in tobacco by ethylene (Sui et al. 2019).

*P*_*MusaCOR2-like*_-GUS displayed weak activation in control conditions which then become significant under drought, salinity, cold, ABA and SA applications. Hence, it can be a useful promoter to express genes associated with unwanted side effects linked with strong and constitutive expression. Genes associated with ABA signalling, cell death and stress associated effectors among others can be driven by *P*_*MusaCOR2-like*_ to improve the agronomic traits of plants.

In conclusion, we performed a functional analysis of the 5’-regulatory region of *MusaCOR2-like* by characterizing its tissue-specific activity and stress-inducible activation profiles using transgenic tobacco plants. Stress inducible profiles of *P*_*MusaCOR2-like*_-*GUS* in tobacco plants were corroborated by the time-dependent transcript profiles of *MusaCOR2-like* under the influence of stress and signalling molecules in banana plants. Furthermore, the current study had identified a stress-inducible promoter of secondary metabolic pathway providing insights into cross-talks between stress signalling and alkaloid biosynthesis regulation. The current study also indicated that a stress-inducible regulatory element like* P*_*MusaCOR2-like*_, could be helpful in futuristic studies in engineering crops with broad-spectrum resistance towards biotic and abiotic stress conditions.

## Data Availability

The DNA sequence generated has been deposited in NCBI with accession number OR714617.

## Abbreviations

ABA: Abscisic acid
AKR: Aldo–keto reductase
BAP: 6-Benzylaminopurine
CHR: Chalcone reductase
COR: Codeinone reductase
DMAS: Deoxymugineic acid synthase
EF1α: Elongation factor 1-alpha
GUS: β-D-Glucuronidase
hpt-II: Hygromycin phosphotransferase-II
MeJA: Methyl jasmonate
MGR: Methylecgonone reductase
NAA: Naphthaleneacetic acid
NOR: NAD(P)-linked oxidoreductase
NDCS: NADPH-dependent deoxychalcone synthase
SA: Salicylic acid
T-DNA: Transfer DNA
X-Gluc: 5-Bromo-4-chloro-3-indolyl glucuronide
